# Proton Beam Therapy for Children With Neuroblastoma: Experiences From the Prospective KiProReg Registry

**DOI:** 10.3389/fonc.2020.617506

**Published:** 2021-01-20

**Authors:** Danny Jazmati, Sarina Butzer, Barbara Hero, Dalia Ahmad Khalil, Julien Merta, Christian Bäumer, Gina Plum, Jörg Fuchs, Friederike Koerber, Theresa Steinmeier, Sarah Peters, Jerome Doyen, Theresa Thole, Matthias Schmidt, Christoph Blase, Stephan Tippelt, Angelika Eggert, Rudolf Schwarz, Thorsten Simon, Beate Timmermann

**Affiliations:** ^1^ Department of Particle Therapy, University Hospital Essen, West German Proton Therapy Centre Essen (WPE), West German Cancer Center (WTZ), Essen, Germany; ^2^ Children’s Hospital, University of Cologne, Cologne, Germany; ^3^ Faculty of Physics, TU Dortmund University, Dortmund, Germany; ^4^ Department of Particle Therapy, University Hospital Essen, West German Proton Therapy Centre Essen (WPE), West German Cancer Center (WTZ), German Cancer Consortium (DKTK), Essen, Germany; ^5^ Department of Pediatric Surgery and Pediatric Urology, University Children’s Hospital Tuebingen, Tübingen, Germany; ^6^ Department of Radiology, University of Cologne, Cologne, Germany; ^7^ Department of Pediatric Oncology/Hematology, Charité-Universitätsmedizin Berlin, Berlin, Germany; ^8^ Department of Nuclear Medicine, University Hospital of Cologne, Cologne, Germany; ^9^ Anästhesienetz Rhein Ruhr, Bochum, Germany; ^10^ University Hospital of Essen, Paediatrics III, Paediatric Haematology and Oncology, Essen, Germany; ^11^ Department of Radiotherapy and Radiooncology, Outpatient Center, University Medical Center Hamburg-Eppendorf, Hamburg, Germany

**Keywords:** neuroblastoma, proton beam therapy (PBT), radiotherapy—adverse effects, pediatric radiation oncology, childhood cancer, retroperitoneal tumor, survival

## Abstract

**Objective:**

Radiotherapy (RT) is an integral part of the interdisciplinary treatment of patients with high-risk neuroblastoma (NB). With the continuous improvements of outcome, the interest in local treatment strategies that reduce treatment-related side effects while achieving optimal oncological results is growing. Proton beam therapy (PBT) represents a promising alternative to conventional photon irradiation with regard to the reduction of treatment burden.

**Method:**

Retrospective analysis of children with high or intermediate risk NB receiving PBT of the primary tumor site during first-line therapy between 2015 and 2020 was performed. Data from the prospective in-house registry Standard Protonentherapie WPE – Kinder- (KiProReg) with respect to tumor control and treatment toxicity were analyzed. Adverse events were classified according to CTCAE Version 4 (V4.0) before, during, and after PBT.

**Results:**

In total, 44 patients (24 male, 20 female) with high (n = 39) or intermediate risk NB (n = 5) were included in the analysis. Median age was 3.4 years (range, 1.4–9.9 years). PBT doses ranged from 21.0 to 39.6 Gray (Gy) (median 36.0 Gy). Five patients received PBT to the MIBG-avid residual at the primary tumor site at time of PBT according to the NB-2004 protocol. In 39 patients radiation was given to the pre-operative tumor bed with or without an additional boost in case of residual tumor. After a median follow-up (FU) of 27.6 months, eight patients developed progression, either local recurrence (n = 1) or distant metastases (n = 7). Four patients died due to tumor progression. At three years, the estimated local control, distant metastatic free survival, progression free survival, and overall survival was 97.7, 84.1, 81.8, and 90.9%, respectively. During radiation, seven patients experienced higher-grade (CTCAE ≥ °3) hematologic toxicity. No other higher grade acute toxicity occurred. After PBT, one patient developed transient myelitis while receiving immunotherapy. No higher grade long-term toxicity was observed up to date.

**Conclusion:**

PBT was a well tolerated and effective local treatment in children with high and intermediate risk NB. The role of RT in an intensive multidisciplinary treatment regimen remains to be studied in the future in order to better define timing, doses, target volumes, and general need for RT in a particularly sensitive cohort of patients.

## Introduction

Neuroblastoma (NB) is the most common extracranial solid tumor of childhood. It has been identified as a neuroectodermal embryonal malignant tumor affecting the sympathetic nervous tissue. Approximately 50% of all patients are diagnosed already with distant metastases (1). The age of >18 months at diagnosis, the detection of amplification of the oncogene MYCN and the presence of distant metastases are well known risk factors for worse disease control and survival (2). The amplification of the MYCN-gene at least five-fold is found in 20–25% of all NBs (3–5). The different risk groups of NB show a heterogeneous course from spontaneous regression to high mortality. While no indication for radiotherapy (RT) is seen in low risk NBs, RT is an integral part of the interdisciplinary treatment of patients with high-risk disease (6). In addition to RT, the treatment regimen in high-risk NBs includes induction chemotherapy, high-dose chemotherapy including tandem transplant, surgery, and immunotherapy. Despite intensive multimodal therapy, the 5-year survival rate of patients with high-risk NB is below 50% (7). Nevertheless, the treatment of metastatic NB has developed considerably with the use of high-dose chemotherapy and immunotherapy (8–10). With the continuous improvements of prognosis, the interest in local therapeutic strategies that potentially can reduce treatment-related side effects while maintaining high tumor control is increasing. Considering the very young age of the affected children, the position of the tumor close to the radiation sensitive organs, such as kidneys and spinal medulla, and the intensive multi-agent chemotherapy applied prior to radiation, proton beam therapy (PBT) represents a promising alternative to conventional photon irradiation. Planning studies in NB have shown that PBT can considerably reduce the radiation exposure of adjacent healthy tissue, potentially reducing the radiation-induced toxicities (11, 12). Furthermore, there are already clinical data demonstrating the effectiveness and feasibility of PBT for NB patients (13, 14). The current study reports on our experiences when treating patients with NB with special consideration of PBT in an intensive multimodal therapy concept.

## Methods

### Patients

Children with high or intermediate risk NB receiving PBT at a single institution during front-line treatment within the prospective in-house registry (Standard Protonentherapie WPE – Kinder- KiProReg; DRKS00005363) were included in this analysis. The high-risk group included all patients with an age of >18 months or >12 months (depending on the study protocol) at diagnosis presenting either with MYCN amplification or with distant metastases. All patients were discussed within the multidisciplinary German NB study board before starting RT. The decision for PBT was considered individually by the national German NB board, which in addition to representatives of pediatric oncology also includes radiation oncology representing both PBT and conventional RT. PBT was typically preferred in younger patients, larger tumors, sites near critical structures, and central sites. Patients treated for a relapse were excluded from this analysis. For all children, data on patients, tumor, treatment, outcome, and toxicity was collected. The registry was approved by the Institutional Ethical Board of the University Duisburg-Essen.

### Treatment

Overall strategies were applied within or according to the respective national or international protocols and treatment standards, respectively. In general, first line treatment for high risk disease consisted of induction chemotherapy, high-dose myeloablative chemotherapy followed by autologous stem-cell rescue and post-consolidation treatment of either immunotherapy with the antibody ch14.18 or retinoic acid. In all patients receiving high-dose chemotherapy with busulfan/melphalan (BuMel), a period of 60–90 days was respected before irradiation started. Consolidating immunotherapy started 4 weeks after the end of RT.

Prior to the start of RT, a re-staging consisting of functional imaging using 123I-mIBG or fluorine-18 (18F) fluorodeoxyglucose (FDG), and cross-sectional imaging was performed. In the case of tumor site in the vicinity of the kidney, a renal scintigraphy was performed prior to radiation planning. In addition, before PBT planning, a central radiological review was performed regarding the preoperative extension and any residual disease in all patients. Furthermore, the surgery reports were evaluated and discussed with the surgeon in case of any uncertainties. A treatment planning computed tomography (CT) was obtained using 1–2 mm slice thickness for all cases. The planning CT was merged with a planning magnetic resonance imaging (MRI) and the initial, preoperative, and most recent previous diagnostic MRIs and MIBGs whenever available. Immobilization of patients for CT-simulation and treatment was ensured using customized immobilization devices depending on the tumor site and geometry. Patients were individually positioned either in prone or supine position. For patients treated according to the “NB 2004 Trial Protocol for Risk Adapted Treatment of Children with Neuroblastoma” (NB 2004/NB2004-HR) (NCT 00410631; NCT 00526318) (15), the extended (by up to 2 cm = CTV) active residual primary tumor was delineated and a total dose of 36–40 Gray (Gy) was delivered to the respective PTV. For patients treated according to High Risk Neuroblastoma Study 1.8 of SIOP-Europe (HRNBL1) (NCT 01704716) protocol or European Low and Intermediate Risk Neuroblastoma Protocol (LINES) (NCT01728155), the gross tumor volume (GTV) included the preoperative extent of the disease adapted to the current anatomy and extended by up to 1 cm in order to account for microscopic spread (CTV). The respective PTV was irradiated up to 21 Gy. Since October 2018, radiation of the primary preoperative tumor region with 21.6 Gy and a tumor boost for the residual tumors with cumulative 36 Gy became the standard in Germany [Association of the Scientific Medical Societies in Germany (AWMF) registration number: 025/008] and was applied in patients not treated in a clinical trial (16). Additionally, in patients with up to three MIBG positive (at the time of RT) osteomedullary metastases, combined irradiation of these lesions was considered. However, the decision to radiate metastases was made very individually by the national interdisciplinary study board. For all dose concepts, a generic relative biological effectiveness (RBE) factor of 1.1 (relative to that of Co-60) was assumed. Proton doses were expressed in terms of Gy (RBE) [Gy (RBE) = proton Gy X 1.1]. After high-dose chemotherapy with Busulfan/Melphalen, the RT planning goals aimed to reduce the maximum dose to gastrointestinal tract, spinal cord, and lung below 30 Gy (RBE). PBT was applied with either uniform (US) or pencil beam scanning (PBS). Treatment planning was carried out with XiO Version 4.80 (Elekta, Stockholm, Sweden) and RayStation^©^ Versions 4.7 to 7.0 (RaySearch Laboratories, Stockholm, Sweden). Typically, two to three beams were used for treatment planning. A multi-field optimization employing intensity modulation was conducted in PBS delivery mode. Typically, the maximum dose of a field was allowed to exceed its nominal dose, i.e. a field configuration with equal weights, by 30%. In order to address potential uncertainties, a density overwrite of the intestine with the average intestine density and a re-computation of the dose distribution was performed. If an interfractional change of the intestine filling had relevant impact on dose robustness, a robust optimization of the treatment plan was conducted simultaneously on the planning CT with or without overwriting intestine density. In addition, the accuracy of dose computation in heterogeneous anatomical regions was taken into account by the Monte-Carlo dose engine as an integral part of the treatment planning system RayStation (17, 18).

Patient set-up, positioning, and treatment were conducted with a ProteusPlus therapy machine (IBA, Lovain-La-Neuve, Belgium). Position verification was facilitated with laser systems, orthogonal X-ray imaging, and a surface tracking camera system (AlignRT, Vision RT Ltd., London, UK). Corrections of the patient set-up were applied to the patient position system, which supports six degrees of freedom. US fields were applied with the IBA universal nozzle which is attached to a 360° gantry. The nozzle was equipped with a Snout180 supporting up two field-specific brass apertures upstream of a range compensator custom milled from an acrylic glass cylinder. PBS fields were applied with a PBS dedicated nozzle which is attached again to a 360° gantry. The PBS delivery proceeded in a step-and-shoot spot scanning mode. The energy and thus, range of the pencil beams was adjusted with an energy-selection system downstream of an isochronous cyclotron.

Whenever tumors in the abdomen and in the thorax displayed relevant respiratory motion, special management to compensate for interplay effect was provided. The motion during the sessions of these cases was monitored with AlignRT. The set-up margin was expanded particularly in cranio-caudal direction. The size of the additional margin was checked against the AlignRT readings. If the respiratory motion was a major concern, also layered repainting was considered resulting in the repeated application of spot segments with the same proton kinetic energy and downscaled fluence (factor of five). Verification MRIs were done on regular basis during treatment, and if any anatomical changes were detected, a new planning CT was obtained with adaptation of contours and plan.

During PBT, regular consultations by radiation oncologists and pediatric oncologists were provided. If patients were too young to consciously cooperate, pediatric anesthesiologists performed deep sedation with i.v. propofol.

### Adverse Events and Follow-Up

Adverse events were classified according to Common Toxicities Criteria on Adverse Events (CTCAE) version 4.0. Adverse events were recorded before, during RT, after 90 days and then at least once a year according to the prospective in-house registry. All patients were assessed weekly during PBT. During FU, patients underwent clinical examination, evaluation of tumor markers, bone marrow examination, cross-sectional and functional diagnostic imaging.

### Statistical Analysis

Qualitative data was reported as frequency (minimum-maximum and percentage). The cut-off was based on the known cut-off or median. Local recurrence (LR) was used to indicate failure in the irradiated region. Consequently, local control (LC) was determined as the absence of local recurrence. Local metastatic relapse (LMR) was used to describe failure at irradiated metastatic lesions. Accordingly, local metastatic control characterized the absence of failure at irradiated lesions. Progression was defined as any event of tumor growth or relapse. Therefore, progression free survival (PFS) represents the time from diagnosis until any failure, relapse, or death. Distant metastatic failure (DMF) was defined as a metastatic recurrence occurring at a metastatic non-irradiated site. Distant metastatic free survival (DMFS) was defined as the absence of metastatic recurrence. Overall survival (OS) was defined as the time from diagnosis to death. LC, PFS, DMFS, and OS were calculated and graphically illustrated using the Kaplan-Meier method. Patients were censored at the time of the last follow-up if not having any event. All statistical analyses were carried out with the Statistical Package for the Social Sciences (SPSS) Version 16.0 under Windows^®^.

### Patients and Tumor Characteristics

A total of 44 patients (24 male; 20 female) were evaluable for this analysis. The median age at PBT was 3.4 years (range, 1.4–9.9 years). The cohort included 39 children (89.0%) with high-risk disease and five children (11%) with local intermediate-risk disease. Further information on patients and tumor characteristics is displayed in **Table 1**.

**Table 1:** Patient and treatment characteristics.

Characteristics		%
**Sex**	n	
male	24	55%
female	20	45%
**Age at diagnosis**	years	
Median	2,6	
Min	0.1	
Max	8.7	
**Age at start of proton therapy**	years	
Median	3.4	
Min	1.4	
Max	9.9	
**Risk grouping**	n	
high	39	89%
intermediate	5	11%
**nMYC Status**	n	
amplified	29	66%
non-amplified	15	34%
**Induction chemotherapy**	n	
yes	44	100%
no	0	0%
**Resection status**	n	
CME	13	30%
IME	30	68%
none	1	2%
**High-dose chemotherapy**	n	
none	5	11%
BuMel	32	73%
MEC	6	14%
TreoMel	1	2%
**Radiotherapy treatment concept**	n	
36–39.6 Gy to residue	4	9%
21.6 Gy to preop.TU; boost to residue to cum. 36 Gy	33	75%
21 Gy to preop. TU	7	16%
**Median total PBT dose**	Gy	
	36.0	
**Median number of fractions**	n	
	20	
**Consolidation therapy**	n	
immunotherapy	36	82%
retino acid	5	11%
none	3	18%

n, number; GPOH regime, German Society for Paediatric Oncology and Hematology (GPOH) regimen [three N5 (cisplatin, etoposide, and vindesine) and three N6 cycles (vincristine, dacarbacine, ifosfamide, and doxorubicine)], Rapid Cojek (cisplatin, vincristine, carboplatin, etoposide, and cyclophosphamide with subsequent administration of Granulocyte Colony Stimulating Factor); CME, Complete macroscopic excision; IME, Incomplete macroscopic excision; BuMel, Busulphan and Melphalan; MEC, Melphalan, Etoposide, and Carboplatin; TreoMel, treosulfan–melphalan.

### Treatment

Induction chemotherapy was performed either according to the German Society for Paediatric Oncology and Hematology (GPOH) regimen consisting of three N5 (cisplatin, etoposide, and vindesine) and three N6 cycles (vincristine, dacarbacine, ifosfamide, and doxorubicine) (n = 34) (6), according to the SIOPEN protocol with the administration of “rapid COJEC” containing cisplatin, vincristine, carboplatin, etoposide, and cyclophosphamide with subsequent administration of Granulocyte Colony Stimulating Factor (n = 8) or according to the European Low and Intermediate Risk Neuroblastoma Protocol (LINES) which comprises a combination chemotherapy consisting of carboplatin and etoposide as well as cyclophosphamide doxorubicin and vincristine (n = 2).

After induction chemotherapy, high-dose chemotherapy with autologous stem cell rescue was provided to 39 patients (88.6%). For high-dose chemotherapy either busulfan and melphalan (BuMel) (n = 32), melphalan, etoposide, and carboplatin (n = 6) or treosulfan–melphalan (n = 1) was administered.

All but one child (98%) underwent tumor resection before RT. Complete macroscopic excision (CME) was achieved in thirteen patients (29.5%). PBT was performed either according to the NB2004 protocol (n = 4), SIOPEN HRNBL1 protocol (n = 5) SIOPEN LINES protocol (n = 2) or according to the German AWMF guideline (n = 33). Of 31 patients with residual tumor at the time of RT, 25 (80.6%) received a dose of more than 30 Gy (RBE). After RT, 5 children received retinoic acid and 36 children received immunotherapy for consolidation purposes.

### Outcome

**Figure 1:**
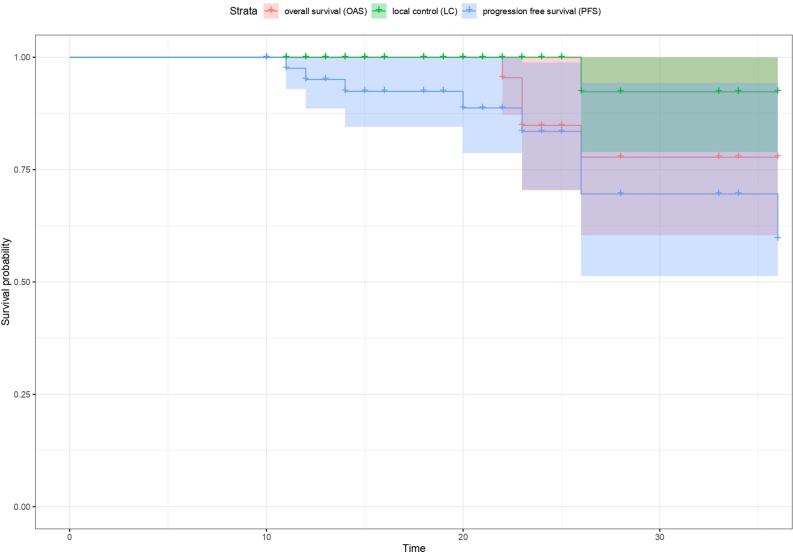
Kaplan-Meier estimates of local control (LC), overall survival (OS) and progression free survival (PFS) respectively for all patients.

After a median FU of 27.6 months from diagnosis, the estimated local control, distant metastatic free survival, progression free survival, and overall survival at 3 years was 97.7, 84.1, 81.8, and 90.9% respectively (**Figure 1**). Out of eight patients with disease progression, one experienced local failure only and seven patients experienced progression with distant metastasis without local failure. Four patients died due to tumor progression. No progression was observed at the irradiated metastatic sites (n = 4).

### Treatment Toxicity

Twenty-three patients presented already at baseline (before starting PBT) with one or more conditions such as veno occlusive disease/sinusoidal obstruction syndrome of the liver (n = 8, 18%), sensorineural hearing loss (n = 5, 11.3%), chronic diarrhea (n = 2, 4.5%), neurological impairment (n = 2, 4.5%), necrosis of the femoral head (n = 1, 2.2%), lung function restriction (n = 1, 2.2%), and grade 3 hematotoxicity (n = 3, 6%). The two patients with chronic diarrhea were later diagnosed with an exocrine pancreatic insufficiency.

No higher grade (CTCAE > °2) acute adverse event was observed during the course of PBT except for hematologic toxicity. Higher-grade hematologic toxicity (> CTCAE ° 2) was reported in seven patients including leukocytopenia (n = 3), thrombocytopenia (n = 3), anaemia (n = 1), and neutropenia (n = 4). One child presented with temporary myelitis (CTCAE °3) associated with impaired leg movement and bladder dysfunction following the first block of immunotherapy after PBT, and was considered as early-delayed complication. The diagnostic imaging displayed a hyperintense MRI change of the spinal cord at the level of the radiation field having received near-maximum dose of 24Gy (RBE). Dosimetric analyses showed no overlap between potential regions with high linear energy transfer (LET) and the area of the myelitis. In one out of five fields applied in two irradiation series, the distal edge stops in the proximity of the myelon. The robustness analysis in terms of stopping-power and set-up uncertainties revealed that even in an unfavorable scenario, the dose received by the myelon would be increased by about 4 Gy (RBE) and would, thus, still be far below the acceptable dose limits. After treatment with corticosteroids and immunoglobin, the clinical symptoms improved.

With regard to long-term toxicity, no symptomatic adverse event was observed attributable to PBT so far. In one patient, an asymptomatic image finding of the irradiated parts of the kidney cortex with normal renal function parameters was observed. 

## Discussion

This study is one of the largest series with standardized data on RT for NB. Our original data displays excellent safety, feasibility and high tumor control gathered from a prospective monoinstitutional registry. Our study complements the existing literature on children undergoing RT for NB confirming high LC and acceptable OS. Local control and survival rates were similar to other studies, which demonstrated a 3- to 5-year LC and OS of 64.7–97% and 35–94% (13, 19–27). In the literature, the most common late effects associated with RT were musculoskeletal abnormalities, gastrointestinal dysfunctions, metabolic disorders, vascular changes, and secondary malignancies (22, 28–31). However, in previous experiences the incidence of RT associated complications was very low, which is consistent with our early results. While the complications of RT in children with NB were only rudimentarily investigated during the early experiences, this study stands out in particular for the close interdisciplinary monitoring and assessment of acute and late effects.

Previously, three comparable studies on PBT for NB have been published. All studies comprised cohorts with similar median age and similar treatment strategies prior to irradiation [9–10, 15]. In all three studies, patients received PBT to the preoperative tumor bed with 21.6–24 Gy (RBE). In two of them, a boost to the residual tumor was administered for a subset of patients. The estimated 3-year local control rate of 97.7% in our study is consistent with the previous PBT experience. Hill et al. reported a local control rate of 97% at 5 years after a median follow-up time of 48.7 months (14). Bagley et al. published on a 5-year local control rate of 87% after a median follow‐up of 60.2 months (13). Lim et al. did not experience any local recurrence in their study but having a limited follow-up time of only 14.9 months. As the characteristics of our cohort, and our results are in line with the previous investigations, it confirms that high local control rates can be achieved with PBT in a very sensitive cohort of very young NB patients (32).

PBT was used less frequently in NB compared to other malignancies. First, total doses are relatively low, and second concerns were raised regarding the robustness of PBT plans in the presence of small bowel or diaphragm and lung in or close to the target volume. Protons lose their energy as they pass through matter and are thereby continuously slowing down. The absorbed dose scales with the inverse of the squared velocity giving rise to an enhancement in ionization near the end of the range, called the Bragg peak. The advantageous depth dose distribution allows for conformal dose coverage of the (static) target and a low radiation burden to healthy tissue. On the other hand, the accurate deposition of dose in the depth domain necessitates concepts to deal with possible variations in geometry or density. These have been considered in the treatment planning of the current study by various measures, individually depending on the tumor site, the amount of respiratory motion or the variability of density of the gastrointestinal tract. All these parameters can affect robustness and, thus, carry a risk of over- or underdosing the target volume and possibly compromising tumor control rates. The overall good local control rates of PBT reported here and elsewhere, confirm feasibility and robustness of PBT treatment plans for the treatment of NB. Plan comparison studies have shown that PBT is particularly advantageous for lateralized target volumes to protect the contralateral side (11, 33).

While RT is considered as a key element in the treatment of high-risk NB, the value of radiation for metastases in NB is seen controversially. While limited cohort studies with a comparison group could not find a positive effect (34, 35), institutional studies revealed promising results (25, 36). In our analysis, local tumor control was achieved for all metastases when irradiated. However, RT to metastatic lesions was only offered to children with limited dissemination at the time of RT defined as up to three MIBG-avid lesions.

Although RT is an integral part of the multimodality treatment of high-risk neuroblastoma, the role of RT with regard to the dose concept is still unclear (37). High local control rates were achieved after irradiation of the preoperative tumor bed with 21 Gy (22, 38–40). It was even discussed by Casey et al. whether lower doses could be sufficient as they obtained comparable results for 18 Gy (41). However, the extent of surgery and the response to high-dose chemotherapy were not taken into account at the radiation planning, so far. There is an on-going controversy about the impact of a macroscopically complete resection compared to an incomplete tumor resection in children with high-risk disease. Analyses of the German NB study NB97 did not show any significant difference for LC, PFS, and OS between the two groups (42). In contrast, the results of the HR-NBL1/SIOPEN study in patients with metastatic NB responding to induction therapy were recently published and showed a small but due to the high number of patients significant improvement for survival and local control rates after macroscopic complete resection (43). In our study, CME was only achieved in 13 patients. Still, only one child experienced local progression. However, out of 31 children with residual tumor at time of RT, 25 children received a dose higher than 30 Gy (RBE) for the residual tumor. In Europe, there is a debate, if, after subtotal resection a dose increase with 36 Gy may be advantageous. A retrospective study from the Memorial Sloan Kettering Cancer Centre showed that patients with residual tumor who had received a dose of more than 30 Gy remained free of local failure (100%), while 30% of those who were irradiated with a dose below 30 Gy experienced local failure (44). Simon et al. had comparable results for patients with residual tumor who received a dose of 36–40 Gy. The local control rate at 3 years in NB 97 for patients with residual tumor who underwent RT was 100% (45). In contrast, the preliminary results of the prospective American ANBL0532 study published recently displayed no benefit of a boost. In this study, 133 patients received preoperative tumor bed irradiation at 21.6 Gy and a boost to the residual tumor up to 36 Gy. No superior results for LC, EFS, or OS at 5 years were achieved when compared to the COG A3973 (NCT 00004188) study. After amendment, patients received only 21.6 Gy radiation of the tumor bed without any boost to the residue (46). The recently opened European collaborative study HR-NBL2 currently investigates the role of a boost after incomplete resection in a randomized fashion. These data are particularly relevant as there is evidence that a dose increase above 30Gy may also increase the likelihood of complications (31).

In contrast to these data but in line with other proton studies, we did not observe relevant, higher grade toxicity, although most of the patients received total doses above 30Gy.

The use of myeloablative chemotherapy (8, 9) and particularly those containing BuMel (47) has been shown to improve the outcome of high-risk NB patients. However, an increased radiosensitivity was assumed after the administration of Busulfan containing myeloablative chemotherapy regimens. Unfortunately, the data on the combination of BuMel and irradiation is very limited and mainly restricted to Ewing sarcomas. Therefore, dose limits applicable to NBs are difficult to define. Seddon et al. reported on a 17-year-old boy presenting with myelopathy after radiation therapy (maximum Dose to myelon: 50.2 Gy) and BuMel (48). Carrie et al. described morbidity-relevant gastrointestinal (GI) side effects (obstruction) after BuMel and pelvic irradiation (49). Bölling et al. evaluated complications attributable to RT after BuMel in the EuroEwing 99 trial with regard to the GI tract, lung, and spinal cord. After a mean follow-up of 7 months, 18 patients being examined did not show any spinal cord complication. After a short mean FU of 1 month, one out of five patients presented with lung dysfunction grade III after irradiation. However, this patient already had pulmonary dysfunction before RT. No higher grade adverse event regarding GI was reported (50). In another report of the EuroEwing 99 study, Whelan et al. presented a case with myelopathy after BuMel and irradiation (51). In our cohort, we had homogenously respected an interval of 60 to 90 days between BuMel and irradiation. In addition the maximum dose to GI tract, lung, and spinal cord was limited to 30 Gy (RBE). With this strategy, we have not observed any complication attributable to the combination of BuMel and RT. Another concern may be raised, regarding the tolerability of RT. Dinutuximab, a monoclonal antibody targeting the glycolipid antigen disialoganglioside expressed on NB cells, has been shown to promote a survival benefit in patients with high-risk NB (52). It is currently unclear whether the combination of RT and immunotherapy may induce additional acute or long term toxicity due to overlapping toxicity profiles (53). Ding et al. reported three patients with myelitis shortly after initiation of dinutuximab therapy and hypothesized a combined effect between irradiation and immunotherapy. The authors postulated that the influence of RT to the blood-brain barrier could have increased the permeability of dinutuximab. Also in our series, one patient presented with a transient myelitis after the first block of dinutuximab following RT also visible within the MRI of the spinal canal related to the radiation field. Fortunately, the symptoms improved after administration of steroids, comparable to the cases presented by Ding et al. [36]. In general, we have to consider RT in NB as particularly challenging in the context of a very demanding and intense multimodal therapy concept.

The proximity of the target volume to the kidney makes nephrotoxicity a significant concern in many patients, particularly when higher RT doses have to be administered. Since residual tumor tissue often remains in the preaortic region close to the large vessel and the kidneys, there is a risk of relevant dose exposure to the kidneys in many patients. In order to protect the better kidney, a split renal function scintigraphy should be performed prior to radiation planning. With this approach we did not observe any clinical relevant radiation induced nephrotoxicity, up to date. Nevertheless, in one patient MRI identified a partial post-RT fibrosis of the kidney cortex as result of partial radiation exposure of the kidney without any subsequent clinical and biochemical evidence of global renal impairment. Interestingly, this patient was operated prior to induction chemotherapy. Therefore, the target volume was based on the initial tumor extension but not on the surgical bed after response to induction chemotherapy generating a very large radiation field.

In the present study, we have to recognize some limitations. The follow-up time is still short and any findings on long-term toxicity cannot be considered representative. Furthermore, patients included in our analysis were irradiated within different study protocols. Finally, we critically acknowledge the retrospective nature of our analysis and the restricted cohort size.

In summary, PBT is a highly conformal RT modality potentially improving the treatment burden. According to our data, PBT for children with NB is feasible with very little acute and early late toxicity. Tumor control rates were high, both for primary disease and metastases. However, results have to be confirmed in larger cohorts and with longer follow-up periods. Any RT in NB patients has to be part of an intensive multidisciplinary treatment regimen and will need intensive investigation with regard to oncological benefit and risk for adverse events.

## Data Availability Statement

The raw data supporting the conclusions of this article are provided by the authors without reservation, in compliance with data protection guidelines.

## Ethics Statement

The studies involving human participants were reviewed and approved by the ethics committee of the University Duisburg Essen. Written informed consent to participate in this study was provided by the participants’ legal guardian/next of kin. Written informed consent was obtained from the minor(s)’ legal guardian/next of kin for the publication of any potentially identifiable images or data included in this article.

## Author Contributions

DJ, SB, BH, DA-K, JM, CBä, GP, JF, FK, TSt, SP, JD, TT, MS, CBl, ST, AE, RS, TSi, and BT contributed to the design and implementation of the research. DJ, SB, BH, DA-K, JM, CBä, GP, JF, FK, TSt, SP, JD, TT, AE, RS, TSi, and BT contributed to the analysis of the results and to the writing of the manuscript. All authors provided critical feedback and helped shape the research, analysis, and manuscript. All authors contributed to the article and approved the submitted version.

## Conflict of Interest

The authors declare that the research was conducted in the absence of any commercial or financial relationships that could be construed as a potential conflict of interest.
